# Overexpression of Human GATA-1 and GATA-2 Interferes with Spine Formation and Produces Depressive Behavior in Rats

**DOI:** 10.1371/journal.pone.0109253

**Published:** 2014-10-23

**Authors:** Miyeon Choi, Sung Eun Wang, Seung Yeon Ko, Hyo Jung Kang, Seung Yeun Chae, Seung Hoon Lee, Yong-Seok Kim, Ronald S. Duman, Hyeon Son

**Affiliations:** 1 Department of Biochemistry and Molecular Biology, College of Medicine, Hanyang University, Seoul, Korea; 2 Graduate School of Biomedical Science and Engineering, Hanyang University, Seoul, Korea; 3 Department of Life Science, Chung-Ang University, Seoul, Korea; 4 Laboratory of Molecular Psychiatry, Center for Genes and Behavior, Departments of Psychiatry, Neurobiology, and Laboratory Medicine, Yale University School of Medicine, New Haven, Connecticut, United States of America; Creighton University, United States of America

## Abstract

Functional consequences to which vertebrate GATA transcription factors contribute in the adult brain remain largely an open question. The present study examines how human GATA-1 and GATA-2 (hGATA-1 and hGATA-2) are linked to neuronal differentiation and depressive behaviors in rats. We investigated the effects of adeno-associated viral expression of hGATA-1 and hGATA-2 (AAV-hGATA1 and AAV-hGATA2) in the dentate gyrus (DG) of the dorsal hippocampus on dendrite branching and spine number. We also examined the influence of AAV-hGATA1 and AAV-hGATA2 infusions into the dorsal hippocampus on rodent behavior in models of depression. Viral expression of hGATA-1 and hGATA-2 cDNA in rat hippocampal neurons impaired dendritic outgrowth and spine formation. Moreover, viral-mediated expression of hGATA-1 and hGATA-2 in the dorsal hippocampus caused depressive-like deficits in the forced swim test and learned helplessness models of depression, and decreased the expression of several synapse-related genes as well as spine number in hippocampal neurons. Conversely, shRNA knockdown of GATA-2 increased synapse-related gene expression, spine number, and dendrite branching. The results demonstrate that hGATA-1 and hGATA-2 expression in hippocampus is sufficient to cause depressive like behaviors that are associated with reduction in spine synapse density and expression of synapse-related genes.

## Introduction

There is a rich cross-talk between transcription factors and signaling pathways that regulate neuronal growth and synapse formation [1], [2], and there is extensive evidence that changes in spine morphology couple with synaptic function in neurons [3]. These functional and structural changes in dendritic spines are thought to be the basis for learning and memory in the brain [4], [5]. Consistent with this idea, changes in spine density are seen in several psychiatric disorders that are associated with deficits in social interaction, cognition and memory function [6]. However, how transcriptional regulation ultimately leads to specific alteration of brain function is not fully understood.

GATA-1 is a member of a family of six zinc-finger proteins, which bind to the (T/A)GATA(G/A) consensus sequence and play important roles in cellular differentiation and proliferation [7]. GATA transcription factors were first described for their role in the proliferation of progenitors and in lineage specification during early hematopoiesis [8], [9]. Recently, GATA-1 was shown to exert repressive effects on spine formation in rat cortical neurons [10] and has been implicated in major depressive disorder (MDD) based on evidence that GATA-1 levels are increased postmortem prefrontal cortex and hippocampus of MDD subjects [11].

GATA-1 is expressed at relatively low levels in brain, but another member of the family GATA-2 is expressed at higher levels in mature neurons in mouse and rats [12]–[14]. GATA-2 plays a role in development of the mouse brain [15], [16]. Phylogenic comparison reveals that GATA-1 protein and two of the DNA binding zinc finger domains are fully conserved between human, mouse and rat [17], [18] (see also www.ihop-net.org/UniPub/iHOP). Human GATA-2 shares 98% amino acid sequence similarity to rats and mouse [7], [19], [20] and has identical zinc finger homology among these species. The zinc finger domains of hGATA-1 and hGATA-2 also have high sequence homology of ∼98% (www.ihop-net.org/UniPub/iHOP). These findings indicate that the GATA-1 and GATA-2 are conserved in vertebrates. However, the influence of GATA transcription factors on adult brain, particularly the hippocampus, remains unclear. In addition, the role of GATA-2 in the actions of stress and in depression-like behaviors has not been examined.

In the current study we examined the influence of stress on the expression of GATA-2, and investigated and compared the effects of hGATA-1 and hGATA-2 transcription factors on neurite outgrowth, spine formation, and synapse-related genes that are regulated by these GATA transcription factor members in rat hippocampal neurons. We also examined the influence of hGATA-1 and hGATA-2 on depressive behaviors in rodent models. The studies aim to elucidate the functional relevance of the GATA transcription factors in hippocampal neurons and how these synaptic changes translate to alterations of depressive behaviors.

## Materials and Methods

### Culture of hippocampal primary neurons

Primary hippocampal neurons were prepared and processed as described previously [21]. Hippocampi from embryo day 16.5 Sprague–Dawley rat (Harlan Sprague Dawley, Indianapolis, IN, USA) embryos were used.

### Construction of adeno-associated viral plasmids and viral production

To construct human GATA expression vector, human GATA cDNA was cloned from human cDNA library (Human Fetus Marathon-Ready cDNA, Clontech, Mountain View, CA, USA) by PCR. The following primer pairs were used for GATA cDNA cloning: for GATA-1 cloning, 5′-GCCACCATGGAGTTCCCTGGCCTG-3′, which includes underlined Kozak sequence and spans nucleotides 1–18 of the human GATA-1 cDNA coding sequence (NM002049.3 GI: 183227689); and 5′-TCATGAGCTGAGCGGAGC-3′, which is complementary to nucleotides 1225–1242 of the human GATA-1 cDNA coding sequence. For GATA-2 cloning, 5′-GCCACCATGGAGGTGGCGCCC-3′, which includes underlined Kozak sequence and spans nucleotides 1–15 of the human GATA-2 cDNA coding sequence (NM001145661.1 GI: 224611698); and 5′-CTAGCCCATGGCGGTCA-3′, which is complementary to nucleotides 1427–1443 of the human GATA-2 cDNA coding sequence.

PCR amplifications were performed in 50 µl reaction volumes with 1 µl (10–100 ng fetal cDNA), 4 U Pyrobest DNA polymerase (TAKARA, Tokyo, Japan), 200 µM each dNTP, 0.6 µM each primer, and 1.3 M betaine on a Thermal cycler (TECHNE, Staffordshire, UK). PCR reactions began with a denaturation step at 94°C for 5 min and 10 cycles of Touchdown (TD) PCR followed by 30 cycles of standard PCR were performed. TD PCR conditions were as follows: 10 s at 98°C, 30 s at annealing temperature stepdowns every cycles of 0.5°C (from +68°C to +63°C), and 2 min at 72°C. The annealing temperature for the final 30 cycles of standard PCR was 62°C with denaturation and extension phases as above. Human GATA cDNA obtained from human fetus cDNA library (Clontech, Mountain View, CA, USA) by PCR was subcloned into pGEM-T-Easy vector (Promega, Madison, WI, USA). After confirmation of its sequence by DNA sequencing, cDNA clone was digested by *Eco*RI. The *Eco*RI fragments were blunt-ended with Klenow DNA polymerase (New England Biolabs, Ipswich, MA, USA) and ligated into the *Eco*RV site of modified adeno-associated viral vector encoding enhanced GFP (AAV-eGFP), generating AAV-eGFP-hGATA1 and AAV-eGFP-hGATA2. The virus was generated using a triple-transfection, helper-free method and was purified with a modified version of a published protocol [22].

### Transfection with small hairpin RNA (shRNA)

Rat shRNAs of GATA2 and a control vector were purchased from GeneCopoeia (Rockville, MD, USA). Transfection of shRNAs (100 nM; target sequence, GGATGGCGTCAAGTATCAA) was performed using a Lipofectamine 2000 reagent according to the manufacturer's instructions (Invitrogen, Carlsbad, CA, USA).

### RT-PCR analysis

Total RNA was prepared from cells with Trizol reagent (Life-technologies, Rockville, MD, USA). Reverse-transcription was conducted as previously described [21]. For quantitative real-time PCR (qRT-PCR), PCR was performed on an iCycler iQ Multi-Color Real-Time PCR Detection System (Bio-Rad Laboratories, Hercules, CA, USA). The housekeeping gene GAPDH was used as a control. Expression of each gene was normalized to the amount of GAPDH to calculate relative transcript levels. Normalized expression values were averaged, and average fold changes were calculated. The primer sequences are as follows: GAPDH, forward: 5′- CTCGTCCCATAGACAAAATGGTGAAG-3′, reverse: 5′- AGACTCCACGACATACTCAGCACC-3′; hGATA-1, forward: 5′-CTTGTAGTAGAGGCCGCAGG-3′, reverse: 5′-GCTCTACCCTGCCTCAACTG-3′; hGATA-2, forward: 5′-TACCACAAGATGAATGGGCA-3′, reverse: 5′-TCTCCTGCATGCACTTTGAC-3′; rGATA1, forward: 5′- GATGGAATCCAGACGAGGAA -3′, reverse: 5′- CTCTCCGCAATTCCCACTAC -3′; rGATA2, forward: 5′-CAG CCT CCA GCT TCA CCC CT-3′ reverse 5′-TGA TGA GCG GCC GAT TCT GT-3′; PSD-95, forward: 5′-GACAACCAAGAAATACCGCT-3′, reverse: 5′-GCTTCTAGGGTGTCCGTGTT-3′; GAP-43, forward: 5′-GGCTCTGCTACTACCGATGC-3′, reverse: 5′-CTGTCGGGCACTTTCCTTAG-3′; RAB4B, forward: 5′-AGCCGGGAGACATACAACTC-3′, reverse: 5′-CCATCCTCTCCGGATCTAGT-3′; MAP2, forward: 5′-GCAAAGTAAGCCTGGTGA-3′, reverse, 5′-ATCTAAGGGAAGAGTGAAAC-3′. GAD1, forward: 5′-CAGAAGTGAAAACAAAAGGC-3′, reverse: 5′-AAACGCTCCATAAACAGTCG-3′; vGluT1, forward: 5′-GGCAGTTTCCAGGACCTCCACTC-3′, reverse: 5′-GCAAGAGGCAGTTGAGAAGGAGAGAG-3′.

### Immunocytochemistry and Immunohistochemistry

Cells were fixed as described before [23]. The following primary antibodies were used: mouse monoclonal anti-green fluorescent protein (GFP) (1∶400, Roche Applied Sciences, Mannheim, Germany) and mouse monoclonal microtubule-associated protein-2 (MAP2) (1∶500, Sigma-Aldrich, Saint Louis, MO, USA) antibodies. Cells were placed in goat anti-mouse secondary antibody conjugated to Alexa488 (1∶400, Invitrogen, Carlsbad, CA, USA) or Cy3 (1∶400, Roche Applied Sciences, Mannheim, Germany). Cells were photographed with a confocal microscope (Leica Microsystems, Wetzlar, Germany). Immunohistochemistry in rat brain was conducted as described previously [21].

### Western blot analysis

Protein extracts were prepared as described previously [23]. The blot was probed with mouse monoclonal β-actin (1∶1000, Santa Cruz, CA, USA), rabbit monoclonal anti-GATA-1 (1∶500, Cell Signaling, Danvers, MA, USA), rabbit monoclonal anti-GATA-2 (1∶500, Abcam, Cambridge, UK), rabbit polyclonal anti-postsynaptic density protein 95 (PSD-95) (1∶1000, Abcam, Cambridge, UK), rabbit polyclonal anti-GAP43 (1∶1000, Abcam, Cambridge, UK), mouse monoclonal anti-Tuj1 (1∶1000, Covance, Berkeley, CA, USA), rabbit polyclonal anti-lamin B1 (1∶1000, Abcam, Cambridge, UK), mouse monoclonal anti-GFP (1∶500, Santa Cruz, CA, USA) followed by treatment goat with anti-mouse or anti-rabbit IgG conjugated with peroxidase (1∶1000, Santa Cruz, CA, USA). Bands were visualized with an ECL detection kit (GenDEPOT, TX, USA).

### Spine density and dendritic arborization analysis

Images were acquired through Z-stacks, which typically consisted of 10 scans at high zoom at 1-µm steps in the z axis. For analysis of spine density in cultured hippocampal neurons, dendrite segments 40 µm in length, from 3 to 5 dendrites per neuron, were sampled from the cell body (proximal) and from end of dendrites (distal). In the hippocampal DG, we focused on second- or third-order dendrites from cells localized in one half of outer molecular layer. For each cell, 3 or more dendritic segments were used for spine analysis. Spine counting was conducted with a 60× objective using the Leica TCS SP5 (Leica Microsystems, Wetzlar, Germany). The number of spines was counted in a 10 µm segment. The final value was averaged from three rats per group and expressed as the number of spines/10 µm. Dendritic branching was investigated by Sholl analysis [24]. A transparent grid showing concentric circles was placed over the dendritic image, the smallest circle was centered in the soma and the distance between each circle was equivalent to 10 µm. Total dendritic length was estimated by counting the total number of circle intersections, and the density of dendrites was defined by counting the number of intersections on each circle. The confocal images of neurons were analyzed using Image J software (http://rsbweb.nih.gov/ij/) and the Sholl analysis plug-in.

### Transcription factor binding motif search

The TFSEARCH (http://www.cbrc.jp/research/db/TFSEARCH.html) was used for the binding motif search in the promoter regions of candidate genes. The transcription factor binding sites were determined in the set of promoters with the parameter of exact matches of core sequences (threshold score = 90.0 as default value cut off) in the vertebrate matrix.

### Animals, stereotaxic surgery and infusions

Male Sprague-Dawley rats (175–250 g) were pair-housed and maintained in standard conditions with a 12-hour light-dark cycle and ad libitum access to food and water. All animal experiments were approved by the Institutional Animal Care and Use Committee of Hanyang University and were performed in accordance with relevant guidelines and regulations. Stereotaxic surgery and infusions were conducted as previously described [21]. Rats were anesthetized with xylazine (6 mg/kg, i.m., Lloyd laboratories, Shenandoar, IA) and ketamine (80 mg/kg i.m., Fort Dodge Animal Health, Overland Park, KS). Bilateral viral injections were performed with coordinates −4.1 mm (anterior/posterior), ±2.4 mm (lateral), and −4.1 mm (dorsal/ventral) relative to the bregma.

### Acute and chronic unpredictable stress (CUS) procedure

For acute stress, rats were individually restrained for a 2 h session in hemicylindrical (20.5×9×6 cm), well-ventilated, Plexiglas tubes. Animals were subsequently killed by rapid decapitation at 2 h after the onset of stress [25]. Ketamine was injected 30 min prior to the onset of stress. Control animals were decapitated immediately after removal from their home cages (total time lapse from removal from cage to decapitation: 1–3 min). CUS is an experimental procedure in which animals are exposed to a variable sequence of mild and unpredictable stressors. Our CUS procedure has been successfully used in the laboratory to produce behavioral changes. The CUS animals were subjected to the same 10 stressors (2 per day) described in Li *et al* [26], for a total of 21 days. Rats were then were treated with vehicle or ketamine (10 mg/kg, i.p.) on day 21 of CUS treatment 24 prior to sacrifice.

### Forced swim test

A forced swim test (FST) was conducted as previously described [21]. Behavioral tests were analyzed by an experimenter blinded to the study code.

### Learned helplessness paradigm

The learned helplessness procedure was performed in commercial shuttle boxes divided into two equal compartments by a central barrier (Gemini Avoidance System, San Diego Instruments, San Diego, CA, USA), as previously described [27]. A computer-operated guillotine door built into the central barrier allowed passage between compartments. On day 1, inescapable footshock (IES) was administered at one side of the shuttle box with the guillotine door closed (60 footshocks, 0.85 mA intensity, 15 sec average duration, 60 sec average intershock interval). Active avoidance testing consisted of 30 trials of escapable footshock (0.65 mA intensity, 35 sec maximum duration, 90 sec average intertribal interval) with the guillotine door open. Each trial used a fixed-ratio 1 schedule, during which one shuttle crossing by rats terminated the shock. Shock was terminated automatically if rats did not escape after 35 sec. A computer automatically recorded the number of escape failures.

### Novelty suppressed feeding test and sucrose preference test

A novelty suppressed feeding test (NSFT) and sucrose preference test (SPT) were conducted as previously described [21].

### Locomotor activity test (LMA)

The general locomotor activity of the rats in the open field test was measured by an automatic video tracking system (SmarTrack, Smartech, Madison, WI, USA). Rats were placed in the central part of the square-shaped arena (77 cm×77 cm×25 cm) and allowed to explore it for 10 min. Total distance traveled (locomotion activity) were recorded for 10 min.

### Statistical analysis

Statistical significance was determined by Student's *t*-test. For behavioral results, statistical differences were determined by analysis of the variance (ANOVA; StatView 5, SAS Software). The *F* values and experimental degrees of freedom are included in the legends of the figures. All data were expressed as mean ± s.e.m.

## Results

### Expression of hGATA-1 and hGATA-2 in cultured rat hippocampal neurons

**Figure 1:**
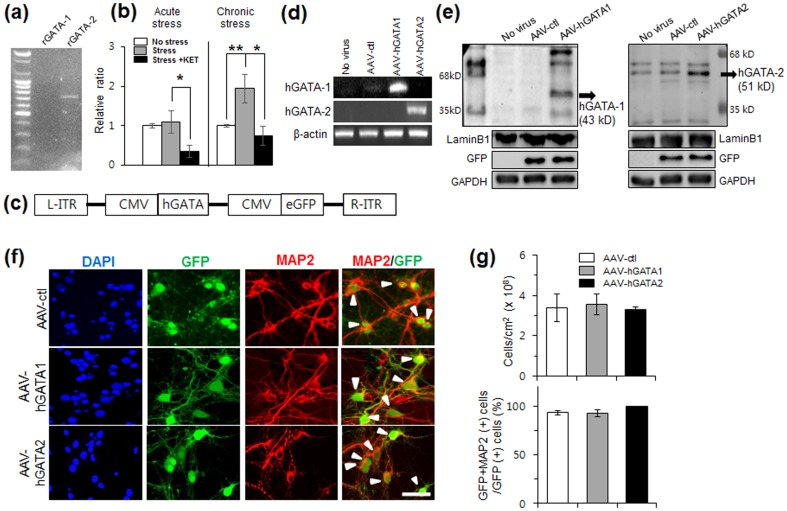
AAV-hGATA-enhanced GFP (eGFP) vector construction and expression in the hippocampal neurons. (a) RT-PCR analysis demonstrating expression of rGATA-2, but not r-GATA-1 in hippocampus. (b) Quantitative RT-PCR analysis of rGATA-2 in the rat hippocampus exposed to acute stress or CUS in the presence or absence of ketamine treatment. Rats were exposed to acute restraint stress for 2 h or CUS for 21 d and received either saline or ketamine (Stress+KET), and sections were subjected to quantitative RT-PCR. Results are expressed as a ratio of nonstressed controls and are the mean ± SEM, each analyzed in triplicate brain sections. rGATA-2 mRNA was increased in acute and CUS animals compared with nonstressed animals (*P*<0.01). CUS animals injected with KET showed a decrease in rGATA-2 mRNA compared with CUS animals (*P*<0.05; *n* = 6 animals in each group). (c) L-ITR and R-ITR, left and right inverted terminal repeats, respectively; CMV, cytomegalovirus promoter. (d) RT-PCR of cDNA isolated from cultured hippocampal neurons transfected with AAV-hGATA1 and AAV-hGATA2. (e) Western blotting analysis was conducted to examine the expression of hGATA transcription factors, and representative blots were shown. The abundance of hGATA-1 (43 kD) and hGATA-2 (51 kD) protein in the nuclear extracts is increased in response to infection with AAV-hGATA1 or AAV-hGATA2. Nuclear extracts were determined by lamin B1, a nuclear marker. (f) Images of hippocampal neurons infected with AAV and stained with antibodies to GFP and MAP2. (g) The total number of cells was revealed by nuclear staining with DAPI. Transfection with AAV-hGATA1 or AAV-hGATA2 showed the same fraction of GFP(+) cells colabeled with MAP2 compared with transduction with AAV-ctl (*P*>0.05). The percentage of MAP2(+) cells among GFP(+) cells is shown as a fraction of the total GFP number of cells. Total MAP2(+) and GFP(+) cells (arrowheads) were counted with a microscope in 10 non-overlapping fields per well. Values represent mean ± s.e.m. from five independent experiments. Scale bar, 50 µm.

We first analyzed expression of rat GATA-1 and -2 (rGATA-1 and -2) in hippocampus. Figure 1a shows mRNA expression of rGATA-2. In contrast, rGATA-1 was not expressed at detectable levels in the hippocampus. Since the involvement of GATA-2 in cellular and behavioral abnormalities related to stress and depression remains an open question, we investigated the influence of acute and chronic stress on the expression levels of rGATA-2 mRNA in the hippocampus. Rats were exposed to acute restraint stress or chronic unpredictable stress (CUS), which results in anhedonic behavior that is reversed with antidepressant treatment, including the rapid acting agent ketamine [27]. While acute restraint had no effect, CUS exposure significantly increased levels of rGATA-2 mRNA in the hippocampus (Fig. 1b, c; *P*<0.01 compared with the nonstressed control). A single dose of ketamine (10 mg/kg) significantly decreased GATA-2 mRNA in acutely stressed rats, and completely reversed the effects of CUS (Fig. 1b, c; *P*<0.05).

Infection of cultured rat hippocampal neurons with AAV-hGATA1-eGFP or AAV-hGATA2-eGFP (Fig. 1a) induced an increase in the expression of hGATA-1 and hGATA-2 by RT-PCR, respectively, compared to control virus (vector only) as expected (Fig. 1b). Western blot analysis of nuclear extracts of the hippocampal neurons demonstrated that levels of hGATA-1 and hGATA-2 protein levels were also increased in response to AAV-hGATA1 and AAV-hGATA2 infection, respectively, compared to control virus (Fig. 1c). The majority of the cells transfected by AAV (with or without hGATA-1 and hGATA-2) differentiate into neurons as determined by the neuronal marker MAP2 (Fig. 1d, e). These experiments show that the hGATA proteins were produced in hippocampal neuronal cells. Infection of cells with AAV-hGATA1 or AAV-hGATA2 resulted in a similar total cell number compared to control virus (Fig. 1e), suggesting that cell survival was not affected by hGATA overexpression.

### Dendritic length and spine density in rat hippocampal neurons

**Figure 2:**
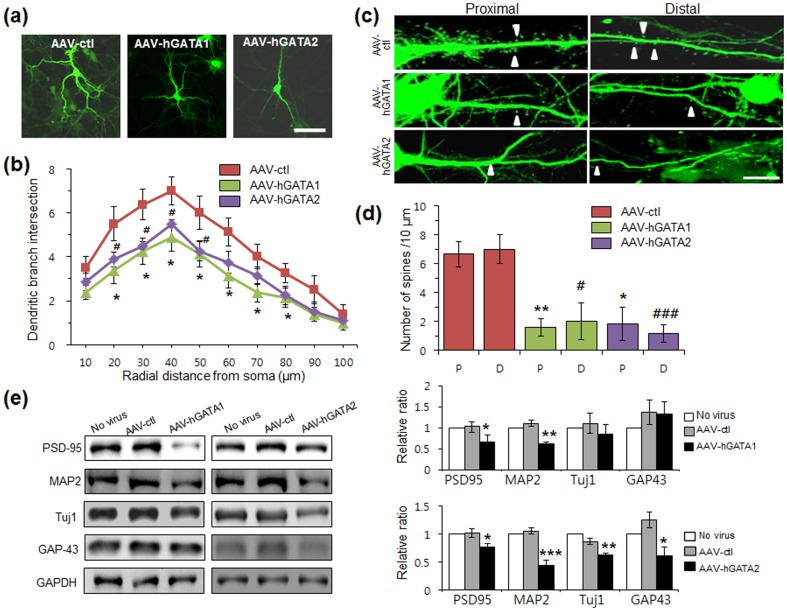
hGATA1 and hGATA-2 decreases dendritic arborization and spine density. Neuronal complexity in the hippocampal neurons transfected with AAV-hGATA1 or AAV-hGATA2 was analyzed based on three-dimensional projections of confocal stack images through complete neuritic extension of GFP(+) process. The neurons were transfected with AAV on 4 DIV and analyzed on 24 DIV. (a) An averaged image of multiple confocal planes across GFP(+) cells. (b) Sholl analysis of GFP(+) cells. Cells transfected with AAV-ctl presented neuritis with an increased ramification compared with cells transfected with AAV-hGAT1 and AAV-hGATA2 (^*^ *P*<0.05). (c) Representative images are shown of high-magnification Z-stack projections of dendrites of GFP(+) cells from AAV-ctl- and AAV-hGATA-transfected cells. A continuous stretch of dendritic processes was imaged. (d) The spine density (arrowheads) of proximal (P) and distal (D) segments was significantly decreased in AAV-hGATA-transfected cells compared to AAV-ctl-transfected rats (^*^ *P*<0.05, ^**^ *P*<0.01 compared to proximal region of AAV-ctl; ^#^ *P*<0.05, ^###^ *P*<0.001 compared to distal region of AAV-ctl; *n* = 9–12 neurons in each group). The data were expressed as the number of spines per 10 µm. (e) MAP2, PSD-95, Tuj1 and GAP-43 expression in the cells described in (a) determined by Western blot analysis. Expression levels are depicted relative to the level of cells that were not exposed to virus (: no virus) for comparison. (*n* = 3–4 biological replicates per group). ^*^ *P*<0.05, ^**^ *P*<0.01, ^***^ *P*<0.001 compared to control vector. Scale bars: *a*, 30 µm; *c*, 10 µm. Student's *t*-test.

Viral expression of rat GATA1 significantly decreases the number of dendrite intersections in cultured rat cortical neurons, indicating decreased complexity (Kang et al., 2012). To investigate whether hGATA-1 and hGATA-2 can alter complexity of rat hippocampal neurons, we analyzed dendrite morphology by fixing and labeling neurons with a GFP-specific antibody (Fig. 2a), and then measured the number of dendrite intersections using Sholl analysis [28]. Consistent with previous results in cortical neurons, viral expression of hGATA1 decreased the complexity of the dendritic arbor of hippocampal neurons (Fig. 2a). When dendritic length was plotted against the shell distances 10–100 µm, differences between AAV-ctl and AAV-hGATA1 were most marked for the middle 20–80 µm range (Fig. 2b; *P*<0.05). Decreased complexity in processes of GFP(+) cells that were infected with AAV-hGAT2 was greatest in the 20–50 µm range (Fig. 2b; *P*<0.05).

Since we observed a morphological change in the rat primary hippocampal neuronal cells that were transfected with AAV-hGATA1 and AAV-hGATA2, we then measured the spine density of those cells to further characterize the morphological changes. Spines protruding from second order dendrites were assessed separately for the proximal and distal dendrites, and results expressed as the number of spines per µm dendritic length (Fig. 2c, d). Infection of cells with AAV-hGATA1 significantly decreased spine density of proximal and distal dendrites compared to AAV-ctl (Fig. 2d; *P*<0.001). Spines of dendrites in cells transfected with AAV-hGATA2 also showed significant decreases in the proximal and distal segments (Fig. 2d; *P*<0.01). Alterations of spine-synapses are accompanied by regulation of postsynaptic proteins, including PSD-95 [27], [29]. Consistent with this idea, the expression of PSD-95 was decreased in neurons transfected with AAV-hGAT1 and AAV-hGATA2 (Fig. 2e).

We then investigated protein levels of β-tubulin type III (Tuj1) and GAP-43, which are known to play an important role in neuronal differentiation, especially in neurite outgrowth [30]. Western blotting analyses showed a significant reduction in the protein levels of Tuj1 and GAP-43 in neurons transfected with AAV-hGATA2, but not AAV-hGATA1 (Fig. 2e). Protein levels of MAP2, which plays a role in neuronal maturation [23], were decreased by overexpression of hGATA1 or hGATA-2 (Fig. 2e).

### hGATA-1 and hGATA-2 produces depressive behaviors

**Figure 3:**
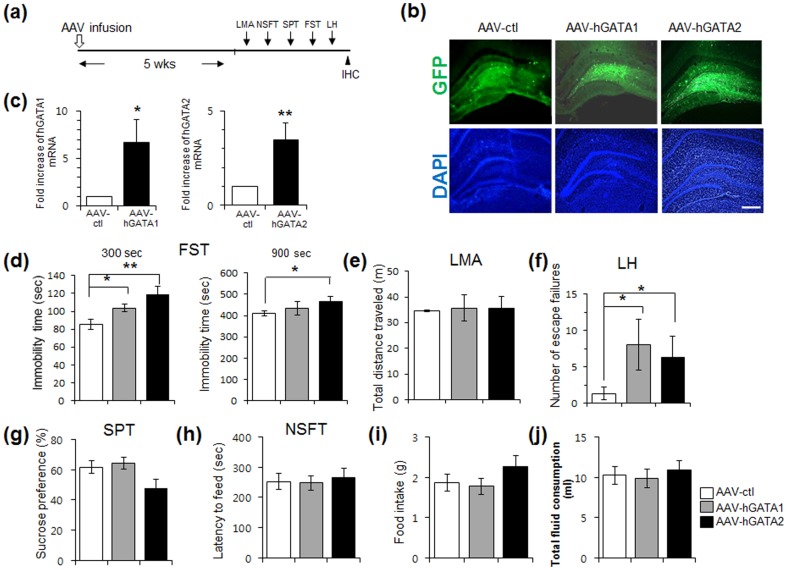
AAV-GATA infusions into hippocampus produce depression-like behavioral actions. (a) Experimental design. Animals were injected with AAV-ctl, AAV-hGATA1 or AAV-hGATA2 and 5 weeks later were tested in behavioral paradigms, and then hippocampal sections were harvested for and immunohistochemistry (IHC) and biochemistry. (b) Representative images of GFP(+) cells in DG from AAV-ctl-eGFP, AAV-hGATA1-eGFP and AAV-hGATA2-eGFP injected animals. (c) Quantitative RT-PCR analysis was conducted to examine the expression of hGATA1 and hGATA-2 mRNA (^**^ *P*<0.01). (d) FST. AAV-hGATA2 rats had a longer immobility score (time in seconds) than AAV-ctl-injected rats during the initial testing for 300 sec (*F*_1,12_ = 11.04, *P* = 0.005) and whole testing session for 900 sec (*F*_1,12_ = 5.12, *P* = 0.042). AAV-hGATA1 rats had a longer immobility score than AAV-ctl-injected rats during the initial testing for 300 sec (*F*_1,12_ = 5.79, *P* = 0.034; 900 sec: *F*_1,12_ = 0.52, *P* = 0.48). (e) Locomoter activity. The total distance moved in the box was similar between groups (*F*_1,10_ = 0.03, *P* = 0.84). (f) LH. AAV-hGATA1 or AAV-hGATA2 transduced rats had more escape failures than AAV-ctl animals (AAV-hGATA1: *F*_1,20_ = 7.09, *P* = 0.028; AAV-hGATA2: *F*_1,19_ = 5.85, *P* = 0.038). (g) SPT. Sucrose preference was not different both in AAV-hGATA1- or AAV-hGATA2-injected animals compared to AAV-ctl-injected animals (AAV-hGATA1: *F*_1,19_ = 0.08, *P* = 0.67; AAV-hGATA2: *F*_1,19_ = 0.19, *P* = 0.78). (h) NSFT. No differences in the latency to feed were shown between AAV-hGATA-injected animals and AAV-ctl-injected animals (AAV-hGATA1: *F*_1,19_ = 0.01, *P* = 0.92; AAV-hGATA2: *F*_1,19_ = 0.09, *P* = 0.76). (i-j) Home cage feeding and total fluid consumption. There was no difference in the home cage food intake (AAV-hGATA1: *F*_1,19_ = 0.08, *P* = 0.76; AAV-hGATA2: *F*_1,19_ = 0.79, *P* = 0.38) and total fluid consumption (AAV-hGATA1: *F*_1,19_ = 0.079, *P* = 0.78; AAV-hGATA2: *F*_1,19_ = 0.19, *P* = 0.66) between AAV-ctl-injected and AAV-hGATA-injected rats. Values represent mean ± s.e.m. from AAV-ctl (*n* = 10), AAV-hGATA1 (*n* = 9–11) and AAV-hGATA2 (*n* = 8–11). Student's *t*-test (c). ANOVA test (d-j). ^*^ *P*<0.05, ^**^ *P*<0.01 compared to control vector. Scale bar: 200 µm.

Given the negative effects of hGATA-1 and hGATA-2 on spine formation and dendritic arborization, we then examined the effects of hGATA-1 and hGATA-2 expression on behaviors in rat models of depression. The AAV vectors were bilaterally injected into the DG of adult rat hippocampus and behavioral testing was conducted 5 weeks after viral infusion (Fig. 3a). This resulted in widespread gene expression in the DG granule cell layer, as shown by viral expression of EGFP (Fig. 3b). Infusions of AAV-hGATA1 and AAV-hGATA2 into the hippocampus also increased levels of hGATA1 and hGATA2 mRNA, respectively, in microdissections of the infused DG area (Fig. 3c). Three models of depression/antidepressant response were tested, including the forced swim test (FST) and learned helplessness (LH), two behavioral despair models, and the sucrose preference test (SPT), a measure of anhedonia, a core symptom of depression [31]. These models are responsive to acute, subchronic, chronic antidepressants, respectively [31]–[33]. Both AAV-hGATA1 and AAV-hGATA2 infusions increased immobility in FST, a pro-depressive response during the initial period (300 sec) of testing (Fig. 3a; *P*<0.05, *P*<0.001, respectively). The depressant response was sustained in rats infused with AAV-hGATA2, but not AAV-hGATA1, during whole testing session (900 sec) (*P*>0.1, *P*<0.05, respectively). Spontaneous locomotor activity (LMA) was not different between these two groups, indicating that the effect observed in FST is not due to general ambulatory differences (Fig. 3e).

In the learned helplessness model, exposing animals to inescapable stress causes escape deficits. In the rats we studied here, infusion of both AAV-hGATA1 and AAV-hGATA2 increased the number of escape failures during the active avoidance testing, without exposure to an inescapable stressor (trials 1–30) (Fig. 3g, *P*<0.05). There were no significant differences in sucrose preference by AAV-hGATA-1 and AAV-hGATA2 compared to AAV-ctl groups (Fig. 3f, *P*>0.05). In addition, both AAV-hGATA1 and hGATA2 had no effect on the latency to feed NSFT, an animal model of anxiety that is responsive to chronic, but not acute antidepressant treatments [34] (Fig. 3h, *P*>0.05 compared to AAV-ctl). Home cage feeding and total fluid consumption were not different between the two groups (Fig. 3i, j). These results show that infusion of AAV-hGATA1 or AAV-hGATA2 produced depressive-like behaviors in two established rat models, the FST and LH.

### hGATA-1 and hGATA-2 repress synapse-related genes

**Figure 4:**
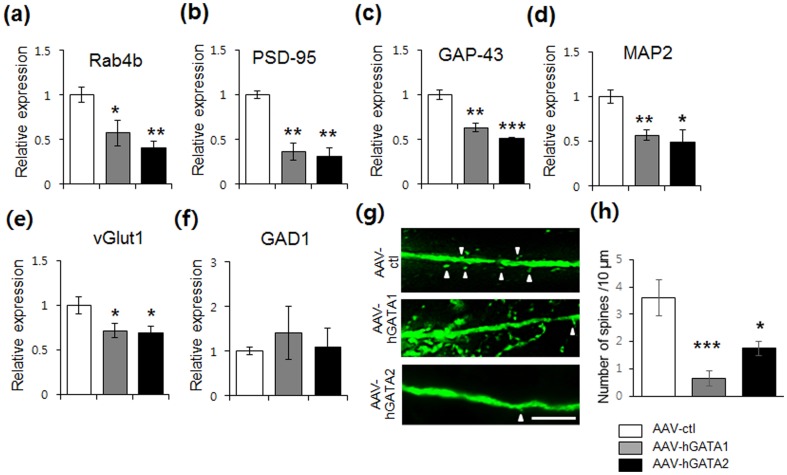
Transcriptional repression of synapse-related genes and a decrease in spine density by AAV-hGATA1 and AAV-hGATA2 in DG of the rat hippocampus. (a–d) Quantitative analysis of synapse-related genes in DG microdissected from rat hippocampus transfected with AAV-hGATA1 or AAV-hGATA2. Rab4b, AAV-hGATA1: *F*_1,4_ = 10.78, *P* = 0.03; AAV-hGATA2: *F*_1,4_ = 46.2, *P* = 0.002; PSD95, AAV-hGATA1: *F*_1,4_ = 31.73, *P* = 0.004; AAV-hGATA2: *F*_1,4_ = 42.8, *P* = 0.002; GAP43, AAV-hGATA1: *F*_1,4_ = 49.8, *P* = 0.002; AAV-hGATA2: *F*_1,4_ = 755.17, *P* = 0.00001; MAP2, AAV-hGATA1: *F*_1,4_ = 46.1, *P* = 0.002; AAV-hGATA2: *F*_1,4_ = 8.3, *P* = 0.04; vGlut1, AAV-hGATA1: *F*_1,4_ = 16.6, *P* = 0.015; AAV-hGATA2: *F*_1,4_ = 24.09, *P* = 0.02; GAD1, AAV-hGATA1: *F*_1,4_ = 0.004, *P* = 0.95; AAV-hGATA2: *F*_1,4_ = 1.59, *P* = 0.27. (g) Representative images are shown of high-magnification Z-stack projections of apical tuft segments of GFP(+) DG granule cells from AAV-ctl, AAV-hGATA1 and AAV-hGATA2-injected rats. (h) Density of dendritic spines (arrowheads) was significantly decreased in AAV-hGATA1- and AAV-hGATA2-injected rats compared with AAV-ctl-injected rats (a main effect of virus, *F*_2,13_ = 12.9, *P*<0.0001; AAV-hGATA1: *F*_1,10_ = 21.15, *P* = 0.0009; AAV-hGATA2: *F*_1,7_ = 5.63, *P* = 0.04). The data were expressed as the number of spines per 10 µm. ANOVA test. ^*^ *P*<0.05, ^**^ *P*<0.01, ^***^ *P*<0.001 compared to control vector. Values represent mean ± s.e.m. of 5–7 cells from three animals per group. Scale bar: 10 µm.

Previous chromatin immunoprecipitation studies have demonstrated that GATA-1 binds to the promoter elements of synapse-related genes, including Rab4b, indicating transcriptional control of these genes [10]. To investigate whether hGATA-1 and hGATA-2 could also regulate the expression of synapse-associated genes in hippocampus, mRNA levels were quantified by RT-PCR. Overexpression of hGATA1 or hGATA-2 in hippocampal DG results in down-regulation of Rab4b mRNA (Fig. 4a, b), a gene implicated in dendritic spine formation, consistent with previous results that elevated GATA-1 underlies the decreased transcription of Rab4b [10]. Given that AAV-hGATA1 and AAV-hGATA2 decrease levels of PSD-95 in cultured hippocampal neurons (Fig. 1a), we examined whether a similar effect is observed in DG transfected by AAV-hGATA1 and AAV-hGATA2. RT-PCR analysis demonstrated that PSD-95 and GAP-43 mRNA levels were decreased in response to AAV-hGATA1 or AAV-hGATA2 infusions into DG (Fig. 4). In addition, overexpression of hGATA-1 or hGATA-2 downregulated the levels of MAP2 mRNA, along with the glutamatergic neuronal marker vesicular glutamate transporter 1 (vGluT1) mRNA (Fig. 4d, e). However, overexpression of hGATA-1 or hGATA-2 had no effects on the differentiation of inhibitory neurons, as determined by quantitative RT-PCR analyzing glutamic acid decarboxylase 1 (GAD1) mRNA, the inhibitory neuronal marker (Fig. 4f). These results indicate that overexpressing hGATA might affect differentiation of glutamatergic neuron subpopulations, although it is possible that altered expression of vGluT1 is related to reduced spine formation. AAV-hGATA1 and AAV-hGATA2 infusion significantly decreased spine density by ∼86% and ∼50%, respectively (Fig. 4g, h, *P*<0.001, *P*<0.05, respectively, compared with the AAV-ctl group).

**Figure 5:**
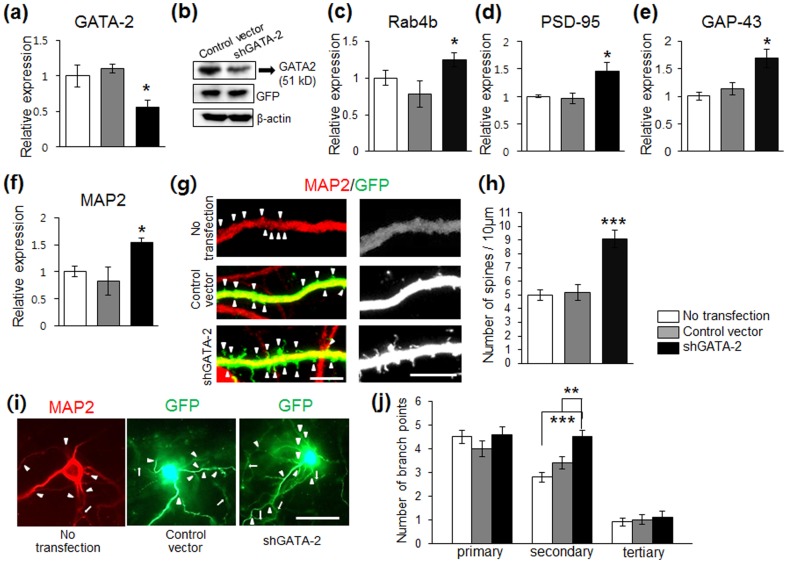
shGATA-2 increases synapse-related genes and spine density. (a–b) Transfection with shGATA-2 caused ∼60% knockdown of GATA2 mRNA and protein expression in rat hippocampal cells. Western blotting analysis was conducted to examine the expression of endogeneous rat GATA-2, and representative blots were shown. The GATA-2 (51 kD) protein in the nuclear extracts is downregulated in response to transfection with shGATA-2 compared to control vector. (c–f) Quantitative analysis of synapse-related genes in rat hippocampal neurons transfected with shGATA-2. Rab4b, *P*<0.05; PSD95, *P*<0.01; GAP43, *P*<0.05; MAP2, *P*<0.05). (g) Representative images are shown of Z-stack projections of dendrites of MAP2(+) and GFP(+) cells from ctl- and shGATA2-transfected cells. A continuous stretch of dendritic processes was imaged. (right panel) high magnification. (h) Density of dendritic spines (arrowheads) was significantly increased in shGATA2-transfected cells compared with controls (^***^ *P*<0.001 compared to controls). The data were expressed as the number of spines per 10 µm. *n* = 5–7 cells. (i) The number of dendritic branches exiting the soma (1′ dendrites), dendritic branches arborizing from 1′ dendrites (2′ dendrites, arrowheads) and dendritic branches arborizing from 2′ dendrites (3′ dendrites, arrows) were counted in neurons immunostained for MAP2 and GFP (*n* = 10–15 cells per treatment). Student's *t* test. ^*^ *P*<0.05, ^**^ *P*<0.01, ^***^ *P*<0.001 compared to control vector. Values represent mean ± s.e.m. Scale bar: 10 µm.

To directly test if synapse-related genes are regulated by GATA, we knocked down endogeneous GATA-2 with small hairpin RNAs (shRNAs) targeted against rat GATA-2 (shGATA-2) in hippocampal neuronal cultures; we did not examine knockdown of GATA-1 due to its low level of endogenous expression under baseline, non stress conditions [10], The transfection of shGATA-2 resulted in approximately 60% knockdown, as shown by real time RT-PCR and Western blot analysis (Fig. 5a, b). When compared to control neurons transfected with control vector alone, significant increases in the expression of Rab4b, PSD-95, GAP-43, MAP2 mRNAs were observed (Fig. 5c, d, e, f) in cells transfected with shGATA-2. The shGATA-2 incubation also significantly increased the spine density (Fig. 5g, h, *P*<0.001, compared with the control group). Regarding dendritic branch points in the MAP2(+) and GFP(+) neurons, we found that cultures differentiated with shGATA-2 contained neurons that had many more local secondary dendritic arborizations than the neurons in the control cultures (Fig. 5i, j, *P*<0.001 and *P*<0.01 compared to no transfection and control vectors, respectively). The shGATA-2 treatment significantly increased the length of spines (Figure S1 in File S1, *P*<0.001, compared with the control group). Together, these results suggest that hGATA-1 and hGATA-2 are involved in transcription of these synapse- and neurite-related genes, and in turn spine-synapse formation.

## Discussion

Here we show that viral expression of hGATA-1 or hGATA-2 transcription factors suppress the expression of genes that are involved in spine formation and neurite outgrowth in the rat hippocampal cells. We also extend our previous studies and demonstrate that chronic stress exposure increases GATA-2 expression in the hippocampus and that this effect is reversed by the rapid acting antidepressant ketamine, supporting a role for GATA-2 in stress related behavioral alterations. Moreover, the results demonstrate that viral expression of hGATA-1 or hGAT-2 in the hippocampus of rat brain decrease in the expression of synapse related genes and cause depressive-like behaviors.

The results of behavioral testing indicate that expression of hGATA-1 and hGATA-2 increases vulnerability to developing ‘depressive-like’ behaviors. Notably, increased hGATA-1 and hGATA-2 levels in the hippocampal DG is sufficient to produce depressive-like effects in the FST and LH, models of behavioral despair and helplessness that are responsive to acute and subchronic antidepressant treatment, respectively. Expression of hGATA-1 or hGATA-2 had no effects on behavior in the SPT and NSFT, models of anhedonia and anxiety. The reasons for the lack of effects in SPT and NSFT are not clear but could be due to the responsiveness of these models to acute (FST), subchronic (LH), or chronic (SPT, NSFT) antidepressant treatments. Alternatively, the differential responses could be related to the role of hippocampus in these behaviors. It would be interesting in future studies to examine the influence of hGATA-1 and hGATA-2 expression in other brain regions, including the prefrontal cortex and amygdala on these behaviors. Nevertheless, the results demonstrate that hGATA-1 and hGATA-2 in the DG of hippocampus produces changes in neuronal morphology that contribute to the expression of depressive-like deficits in tests that assess despair and helplessness, but not anhedonia and anxiety.

Overexpression of GATA-1 in rat cortex has previously been shown to cause depressive behaviors that are associated with alterations of neuronal morphology [10]. Further evidence for a role of GATA1 in depression was provided by a recent genome microarray study of postmortem MDD subjects, which showed that levels of GATA1 mRNA were increased in the DG, but not CA1 subregion of the hippocampus [11]. Accordingly, we examined the influence of GATA transcription factors on the morphology of hippocampal neurons and depressive behaviors. This strategy allows analysis of the overall effects of these transcription factors on synapse related target genes that may occur via conserved GATA promoter sequences. An advantage of this approach is that we are examining the endogenous target genes in their normal chromatin environment in rat hippocampal cells, which express GATA-2, and low levels of GATA-1 [14]. Based on low endogenous levels, viral expression of hGATA-1 may produce a distinct effect on the transactivation of genes. In contrast, because levels of endogenous GATA-2 are relatively high, viral expression of hGATA-2 may produce less physiological changes. However, hGATA-2 protein appears to exhibit a broader binding sequence specificity compared with that of mouse GATA-1 and hGATA-3 [35]. Thus even small changes in the level of hGATA-2 proteins might affect transactivation of GATA-2 responsive genes and in turn produces depressive-like deficits. Our results together with the previous finding [10] indicate that GATA1 induces depressive-like behaviors and a decrease in spine density when it is overexpressed either in the prefrontal cortex or hippocampus. Accordingly, hGATA-2, which produces similar depressive-like behaviors when expressed in hippocampal neurons, might have depressive effects on animal behaviors when it is overexpressed in the prefrontal cortex as well. Given that hGATA-1 and -2 control depressive-like behaviors in rats, one might assume there is a link between hGATA transcription factors and adult hippocampal neurogenesis, a form of neural plasticity that is increased by antidepressant treatments [36]. However, there have been no reports that GATA transcription factors regulate adult hippocampal neurogenesis, and further studies will be needed to address this question.

The results show that some of the synaptic plasticity-related genes normally expressed in neuronal cells respond to hGATA-1 and hGATA-2 transcription factor expression. Interestingly, besides previously known GATA-1 responding genes, PSD-95, GAP-43 and MAP2 showed a response to changes in levels of the hGATA-1/hGATA-2 *in vivo*. Our scans for canonical GATA sites throughout the locus based on aligned DNA sequences indicate that PSD-95, GAP-43 and MAP2 have potential GATA-1 and GATA-2 binding sites (http://www.cbrc.jp/research/db/TFSEARCH.html). Therefore, it appears that genes having conserved GATA sites showed a response to changes in levels of the GATA proteins. Indeed many genes have GATA promoter elements (conserved or not) because of the short recognition sequence (6 to 8 bp), which are frequently represented in genomic DNA (e.g. >50 sites in MAP2 gene) [35], [37]. However, the functional regulation of GATA transcription factor binding *in vivo* is controlled by many different factors. The primary determinants of transcriptional outcome would be the context in which a potential binding site is embedded, local chromatin structure, and synergistic interactions with other transcription factors recognizing neighboring sites. Thus, the repressive effects of hGATA-1 and hGATA-2 on the synapse-related genes might not be solely due to the presence or number of DNA binding sites, but rather is related to the transcriptome environment of genes, which is also influenced by experiential and behavioral factors. Given the high degree of similarity of DNA binding zinc finger sequences between hGATA-1 and hGATA-2, hGATA-2 might be the primary repressor *in vivo* until hGATA-1 is induced by stimuli such as chronic stress. Further studies will be required to address how the mammalian GATA proteins regulate the transcription of synapse-related genes via different binding specificities at different sites *in vivo*.

Given that hGATA-1 and hGATA-2 may regulate spine morphology and synaptic function in hippocampal neurons and depressive behaviors in rats, further studies will be warranted to investigate whether hGATA-1 and hGATA-2 play distinct roles in neurons of the adult brain including hippocampus. Full evaluation of the expression and role of the hGATA-1 and hGATA-2 on the transcription of target genes in rodents, as well as the localization in different populations of neurons in human brain will further elucidate the molecular mechanisms that underlie the pathophysiology and eventually the treatment of stress related illnesses such as depression and related psychiatric disorders.

## Supporting Information

File S1 **Supporting Materials and Methods and Figure S1.**
(DOCX)

## References

[1] Gutierrez H, Davies AM (2011) Regulation of neural process growth, elaboration and structural plasticity by NF-kappaB. Trends Neurosci 34: 316–325. 21459462 10.1016/j.tins.2011.03.001 PMC3115056

[2] Eyre H, Baune BT (2012) Neuroplastic changes in depression: a role for the immune system. Psychoneuroendocrinology 37: 1397–1416. 22525700 10.1016/j.psyneuen.2012.03.019

[3] Kasai H, Matsuzaki M, Noguchi J, Yasumatsu N, Nakahara H (2003) Structure-stability-function relationships of dendritic spines. Trends Neurosci 26: 360–368. 12850432 10.1016/S0166-2236(03)00162-0

[4] Holtmaat A, Svoboda K (2009) Experience-dependent structural synaptic plasticity in the mammalian brain. Nat Rev Neurosci 10: 647–658. 19693029 10.1038/nrn2699

[5] Kasai H, Fukuda M, Watanabe S, Hayashi-Takagi A, Noguchi J (2010) Structural dynamics of dendritic spines in memory and cognition. Trends Neurosci 33: 121–129. 20138375 10.1016/j.tins.2010.01.001

[6] Glantz LA, Lewis DA (2000) Decreased dendritic spine density on prefrontal cortical pyramidal neurons in schizophrenia. Arch Gen Psychiatry 57: 65–73. 10632234 10.1001/archpsyc.57.1.65

[7] Pan X, Minegishi N, Harigae H, Yamagiwa H, Minegishi M, et al (2000) Identification of human GATA-2 gene distal IS exon and its expression in hematopoietic stem cell fractions. J Biochem 127: 105–112. 10731672 10.1093/oxfordjournals.jbchem.a022570

[8] Begley CG, Green AR (1999) The SCL gene: from case report to critical hematopoietic regulator. Blood 93: 2760–2770. 10216069

[9] Shivdasani RA, Orkin SH (1996) The transcriptional control of hematopoiesis. Blood 87: 4025–4039. 8639758

[10] Kang HJ, Voleti B, Hajszan T, Rajkowska G, Stockmeier CA, et al (2012) Decreased expression of synapse-related genes and loss of synapses in major depressive disorder. Nat Med 18: 1413–1417. 22885997 10.1038/nm.2886 PMC3491115

[11] Duric V, Banasr M, Licznerski P, Schmidt HD, Stockmeier CA, et al (2010) A negative regulator of MAP kinase causes depressive behavior. Nat Med 16: 1328–1332. 20953200 10.1038/nm.2219 PMC3066515

[12] Zhou Y, Yamamoto M, Engel JD (2000) GATA2 is required for the generation of V2 interneurons. Development 127: 3829–3838. 10934027 10.1242/dev.127.17.3829

[13] Craven SE, Lim KC, Ye W, Engel JD, de Sauvage F, et al (2004) Gata2 specifies serotonergic neurons downstream of sonic hedgehog. Development 131: 1165–1173. 14973276 10.1242/dev.01024

[14] Wallach I, Zhang J, Hartmann A, van Landeghem FK, Ivanova A, et al (2009) Erythropoietin-receptor gene regulation in neuronal cells. Pediatr Res 65: 619–624. 19218878 10.1203/PDR.0b013e31819ea3b8

[15] El Wakil A, Francius C, Wolff A, Pleau-Varet J, Nardelli J (2006) The GATA2 transcription factor negatively regulates the proliferation of neuronal progenitors. Development 133: 2155–2165. 16672344 10.1242/dev.02377

[16] Nardelli J, Thiesson D, Fujiwara Y, Tsai FY, Orkin SH (1999) Expression and genetic interaction of transcription factors GATA-2 and GATA-3 during development of the mouse central nervous system. Dev Biol 210: 305–321. 10357893 10.1006/dbio.1999.9278

[17] Morceau F, Schnekenburger M, Dicato M, Diederich M (2004) GATA-1: friends, brothers, and coworkers. Ann N Y Acad Sci 1030: 537–554. 15659837 10.1196/annals.1329.064

[18] Gong QH, Stern J, Dean A (1991) Transcriptional role of a conserved GATA-1 site in the human epsilon-globin gene promoter. Mol Cell Biol 11: 2558–2566. 2017165 10.1128/mcb.11.5.2558-2566.1991 PMC360025

[19] Minegishi N, Ohta J, Suwabe N, Nakauchi H, Ishihara H, et al (1998) Alternative promoters regulate transcription of the mouse GATA-2 gene. J Biol Chem 273: 3625–3634. 9452491 10.1074/jbc.273.6.3625

[20] Kumari J, Bogwald J, Dalmo RA (2009) Transcription factor GATA-3 in Atlantic salmon (Salmo salar): molecular characterization, promoter activity and expression analysis. Mol Immunol 46: 3099–3107. 19576635 10.1016/j.molimm.2009.06.008

[21] Son H, Banasr M, Choi M, Chae SY, Licznerski P, et al (2012) Neuritin produces antidepressant actions and blocks the neuronal and behavioral deficits caused by chronic stress. Proc Natl Acad Sci U S A 109: 11378–11383. 22733766 10.1073/pnas.1201191109 PMC3396528

[22] Hommel JD, Sears RM, Georgescu D, Simmons DL, DiLeone RJ (2003) Local gene knockdown in the brain using viral-mediated RNA interference. Nat Med 9: 1539–1544. 14634645 10.1038/nm964

[23] Kim JS, Chang MY, Yu IT, Kim JH, Lee SH, et al (2004) Lithium selectively increases neuronal differentiation of hippocampal neural progenitor cells both in vitro and in vivo. J Neurochem 89: 324–336. 15056276 10.1046/j.1471-4159.2004.02329.x

[24] Sholl DA (1953) Dendritic organization in the neurons of the visual and motor cortices of the cat. J Anat 87: 387–406. 13117757 PMC1244622

[25] Melia KR, Ryabinin AE, Schroeder R, Bloom FE, Wilson MC (1994) Induction and habituation of immediate early gene expression in rat brain by acute and repeated restraint stress. J Neurosci 14: 5929–5938. 7931554 10.1523/JNEUROSCI.14-10-05929.1994 PMC6576983

[26] Li N, Liu RJ, Dwyer JM, Banasr M, Lee B, et al (2011) Glutamate N-methyl-D-aspartate receptor antagonists rapidly reverse behavioral and synaptic deficits caused by chronic stress exposure. Biol Psychiatry 69: 754–761. 21292242 10.1016/j.biopsych.2010.12.015 PMC3068225

[27] Li N, Lee B, Liu RJ, Banasr M, Dwyer JM, et al (2010) mTOR-dependent synapse formation underlies the rapid antidepressant effects of NMDA antagonists. Science 329: 959–964. 20724638 10.1126/science.1190287 PMC3116441

[28] Beauquis J, Roig P, De Nicola AF, Saravia F (2010) Short-term environmental enrichment enhances adult neurogenesis, vascular network and dendritic complexity in the hippocampus of type 1 diabetic mice. PLoS One 5: e13993. 21085588 10.1371/journal.pone.0013993 PMC2981567

[29] Keith D, El-Husseini A (2008) Excitation Control: Balancing PSD-95 Function at the Synapse. Front Mol Neurosci 1: 4. 18946537 10.3389/neuro.02.004.2008 PMC2526002

[30] Denny JB (2006) Molecular mechanisms, biological actions, and neuropharmacology of the growth-associated protein GAP-43. Curr Neuropharmacol 4: 293–304. 18654638 10.2174/157015906778520782 PMC2475799

[31] Banasr M, Duman RS (2008) Glial loss in the prefrontal cortex is sufficient to induce depressive-like behaviors. Biol Psychiatry 64: 863–870. 18639237 10.1016/j.biopsych.2008.06.008 PMC2709733

[32] Hunsberger JG, Newton SS, Bennett AH, Duman CH, Russell DS, et al (2007) Antidepressant actions of the exercise-regulated gene VGF. Nat Med 13: 1476–1482. 18059283 10.1038/nm1669

[33] Banasr M, Chowdhury GM, Terwilliger R, Newton SS, Duman RS, et al (2010) Glial pathology in an animal model of depression: reversal of stress-induced cellular, metabolic and behavioral deficits by the glutamate-modulating drug riluzole. Mol Psychiatry 15: 501–511. 18825147 10.1038/mp.2008.106 PMC3347761

[34] Santarelli L, Gobbi G, Debs PC, Sibille ET, Blier P, et al (2001) Genetic and pharmacological disruption of neurokinin 1 receptor function decreases anxiety-related behaviors and increases serotonergic function. Proc Natl Acad Sci U S A 98: 1912–1917. 11172050 10.1073/pnas.041596398 PMC29356

[35] Merika M, Orkin SH (1993) DNA-binding specificity of GATA family transcription factors. Mol Cell Biol 13: 3999–4010. 8321207 10.1128/mcb.13.7.3999-4010.1993 PMC359949

[36] Malberg JE, Eisch AJ, Nestler EJ, Duman RS (2000) Chronic antidepressant treatment increases neurogenesis in adult rat hippocampus. J Neurosci 20: 9104–9110. 11124987 10.1523/JNEUROSCI.20-24-09104.2000 PMC6773038

[37] Gumina RJ, Kirschbaum NE, Piotrowski K, Newman PJ (1997) Characterization of the human platelet/endothelial cell adhesion molecule-1 promoter: identification of a GATA-2 binding element required for optimal transcriptional activity. Blood 89: 1260–1269. 9028949

