# Supplementary material for: Overexpression of Human GATA-1 and GATA-2 Interferes with Spine Formation and Produces Depressive Behavior in Rats
Source: PLoS One. 2014 Oct 23;9(10):e109253. doi: 10.1371/journal.pone.0109253 (PMC4207676; doi:10.1371/journal.pone.0109253)
Supplement: File S1 — Supporting Materials and Methods and Figure S1. (DOCX) [file pone.0109253.s001.docx]

**Overexpression of human GATA-1 and GATA-2 interferes with spine formation and produces depressive behavior in rats**

Miyeon Choi^1#^, Sung Eun Wang^2#^, Seung Yeon Ko^2#^, Hyo Jung Kang^3#^, Seung Yeun Chae^2^ , Seung Hoon Lee^2^, Yong-Seok Kim^1,2^, Ronald S. Duman^4^ and Hyeon Son^1,2*^

**Supplementary Materials and Methods**

Quantification of dendritic spine length was performed as described [1]. Morphometric measurements were performed with the aid of Leica Application Suite Advanced Fluorescence (LAS AF, online help version 3.3, D-68165 Mannheim, Germany). The length of an individual spine was measured from the tip of the spine head to the interface with the dendritic stalk.

**References**

1. Ji Y, Lu Y, Yang F, Shen W, Tang TT, et al. (2010) Acute and gradual increases in BDNF concentration elicit distinct signaling and functions in neurons. Nat Neurosci 13: 302-309.

**Figure S1.**

**
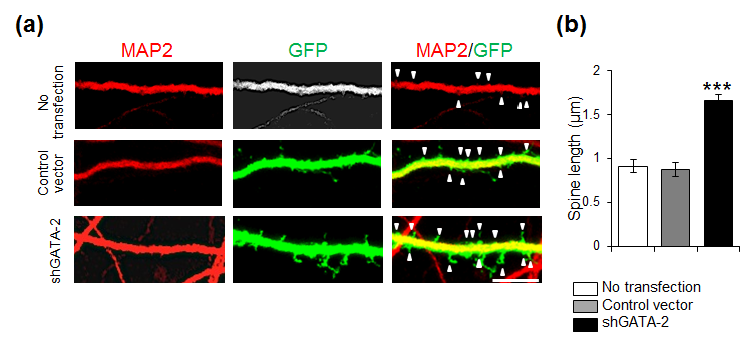
**

**Fig. 1 shGATA-2 increases spine length.** (a-b) Length of dendritic spines (arrowheads) was significantly increased in shGATA2-transfected cells compared with controls (^***^*P* < 0.001 compared to controls). The data were expressed as the length of spines. Student’s *t* test. Values represent mean ± s.e.m. of 10-15 cells. Scale bar: 10 µm.
